# Supplementary material for: Conjugation of Mannans to Enhance the Potency of Liposome Nanoparticles for the Delivery of RNA Vaccines
Source: Pharmaceutics. 2021 Feb 9;13(2):240. doi: 10.3390/pharmaceutics13020240 (PMC7916126; doi:10.3390/pharmaceutics13020240)
Supplement: Supplementary file 1 [file pharmaceutics-13-00240-s001.pdf]

pharmaceutics

MDPI

Supplementary Materials: Conjugation of Mannans to Enhance the Potency of Liposome Nanoparticles for the Delivery of RNA Vaccines

Roshan Goswami [1,2], Derek T. O'Hagan [3], Roberto Adamo [2,*], Barbara C. Baudner [2,*]

NMR spectra of synthesized mannan-cholesterol conjugates

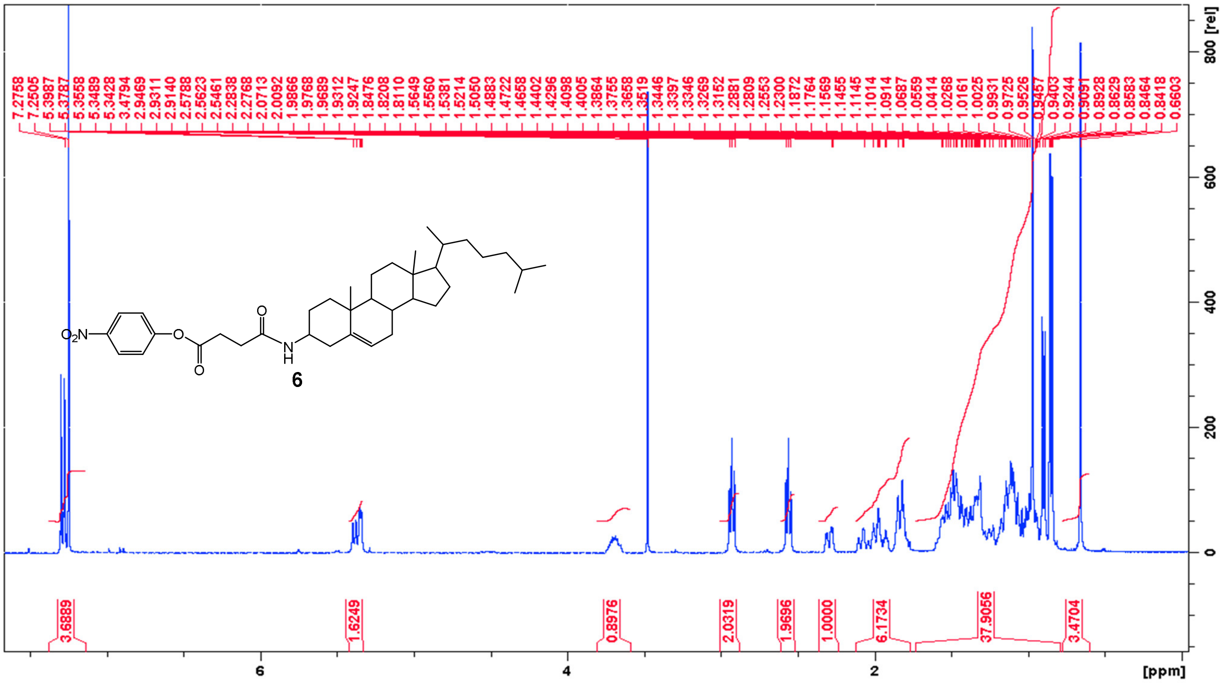

Figure S1. ^{1}H NMR (400 MHz, $CDCl_3$) of the cholesterol derivative **6.**

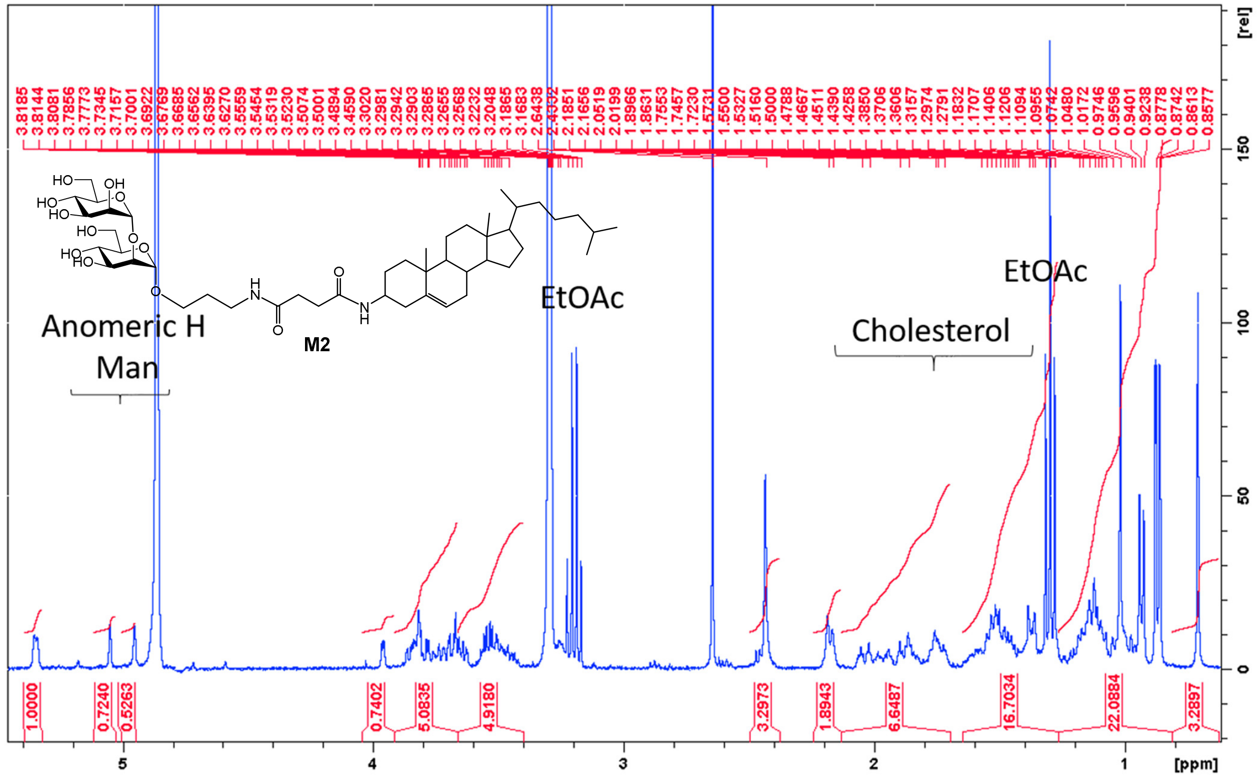

Figure S2. ^{1}H NMR (400 MHz, MeOD) of conjugate **M2**.

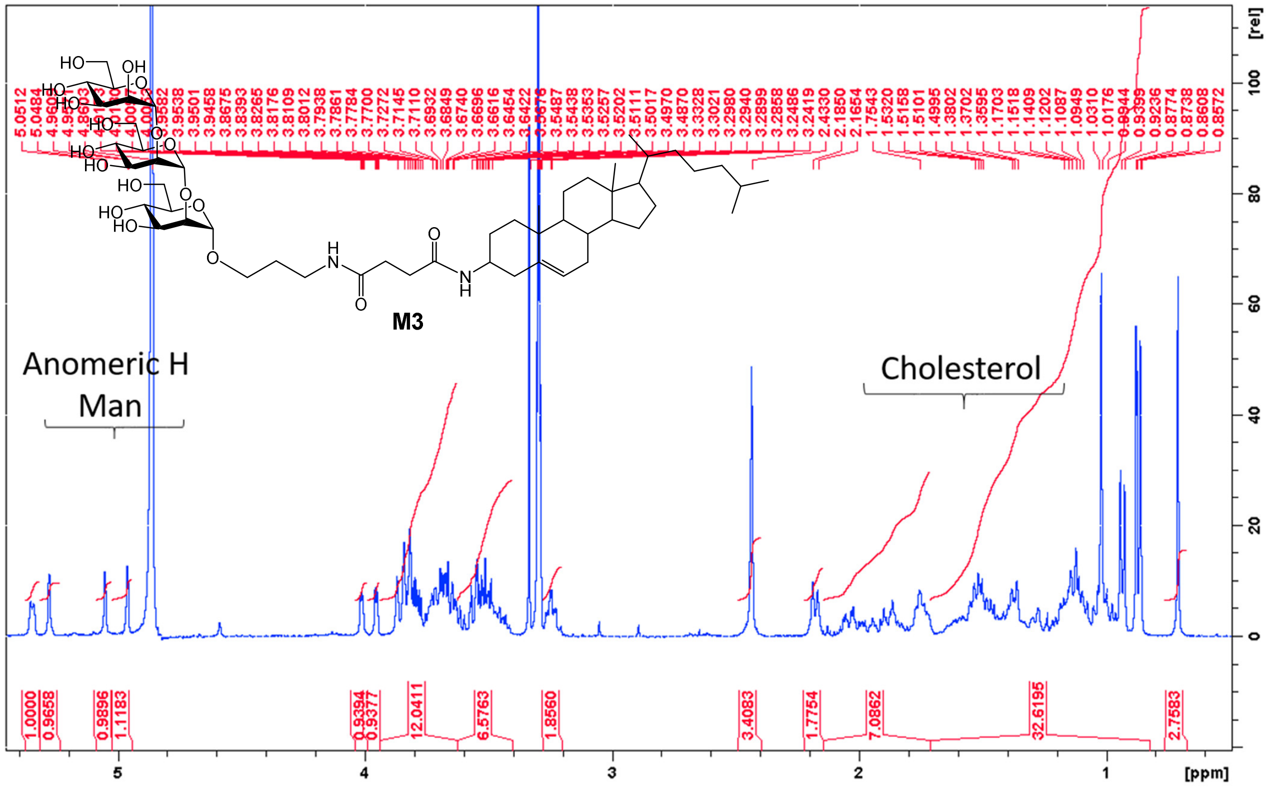

Figure S3. ^{1}H NMR (400 MHz, MeOD) of conjugate **M3**.

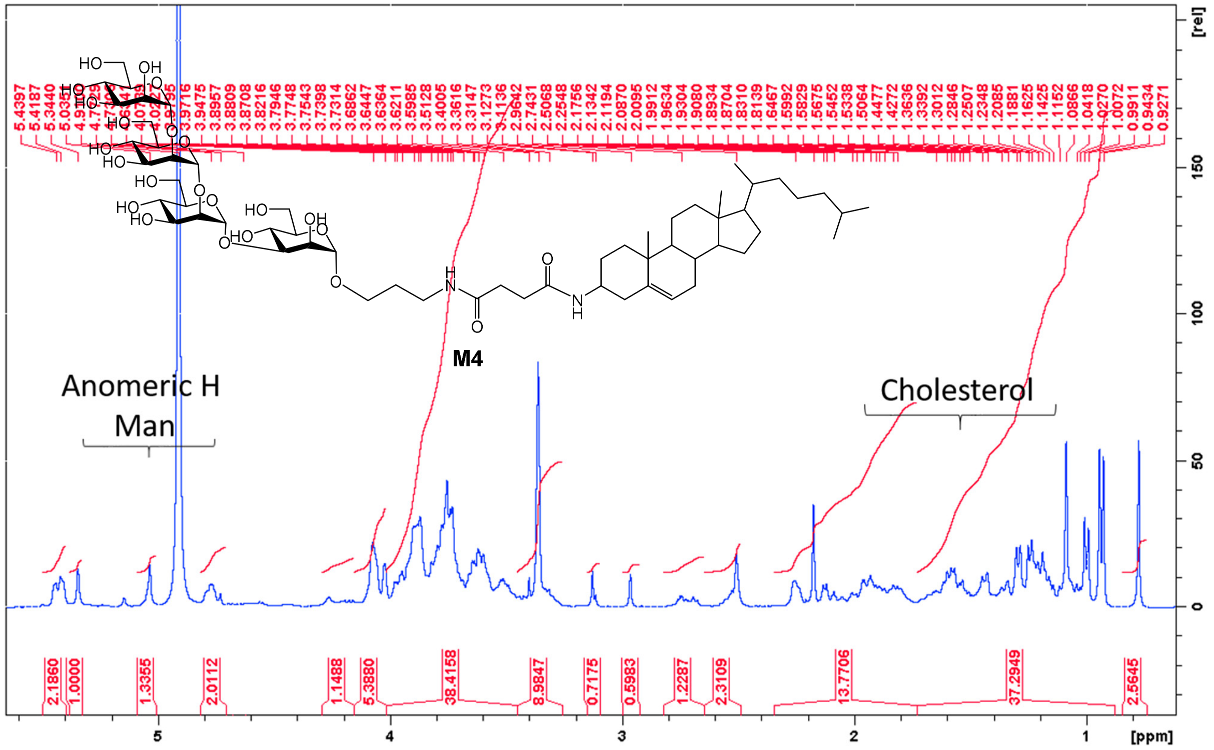

Figure S4. ^{1}H NMR (400 MHz, MeOD) of conjugate **M4**.
